# Supplementary material for: High Multi-Environmental Mechanical Stability and Adhesive Transparent Ionic Conductive Hydrogels Used as Smart Wearable Devices
Source: Polymers (Basel). 2022 Dec 5;14(23):5316. doi: 10.3390/polym14235316 (PMC9739927; doi:10.3390/polym14235316)
Supplement: Supplementary file 1 [file polymers-14-05316-s001.zip › polymers-2067218-supplementary.pdf]

Supporting Information

High Multi-Environmental Mechanical Stability and Adhesive Transparent Ionic Conductive Hydrogels Used as Smart Wearable Devices

Yuxuan Wu, Jing Liu, Zhen Chen, Yujie Chen *, Wenzheng Chen, Hua Li and Hezhou Liu *

State Key Laboratory of Metal Matrix Composites, School of Materials Science and Engineering, Shanghai Jiao Tong University, Shanghai 200240, China

* Correspondence: yujiechen@sjtu.edu.cn (Y.C.); hzliu@sjtu.edu.cn (H.L.); Tel.: +86-21-34202546 (H.L.)

Table S1. Composition of the hydrogels.

Name	TA@BC (wt %)	AA (g)	AMPS (g)	UV initiator (μl)	H_2O (g)	$CaCl_2$ (g)
**P(AA-AMPS)**	0	1.25	1.25	4	10	0
**P(AA-AMPS)-TA@BA$_{0.25}$-Ca^{2+}**	0.25	1.25	1.25	4	10	4.4
**P(AA-AMPS)-TA@BA$_{0.5}$**	0.5	1.25	1.25	4	10	0
**P(AA-AMPS)-TA@BA$_{0.5}$-Ca^{2+}**	0.5	1.25	1.25	4	10	4.4
**P(AA-AMPS)-TA@BA$_1$-Ca^{2+}**	1.0	1.25	1.25	4	10	4.4

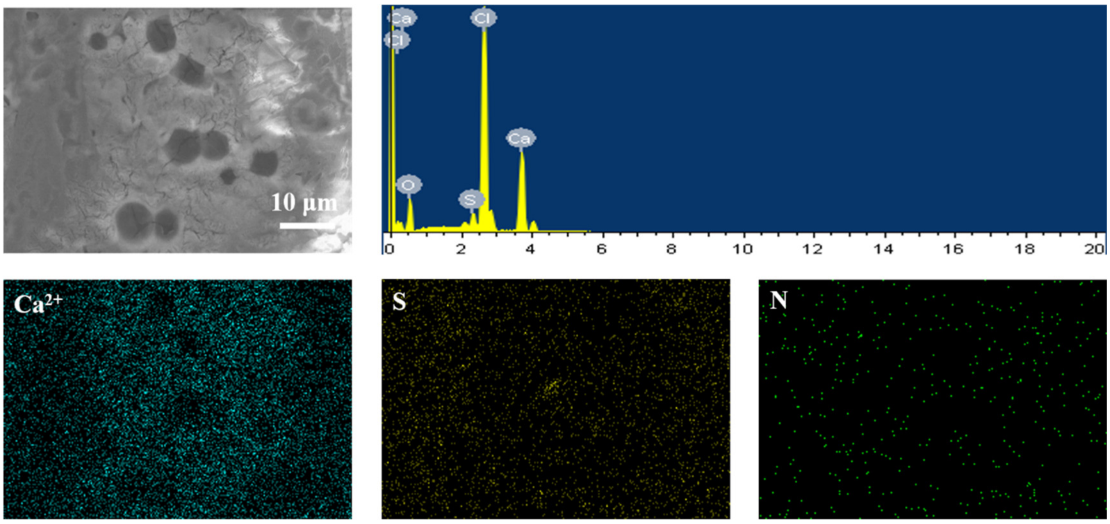

Figure S1. EDS images of the surface of the P(AA-AMPS)-TA@BC-Ca^{2+} hydrogel.

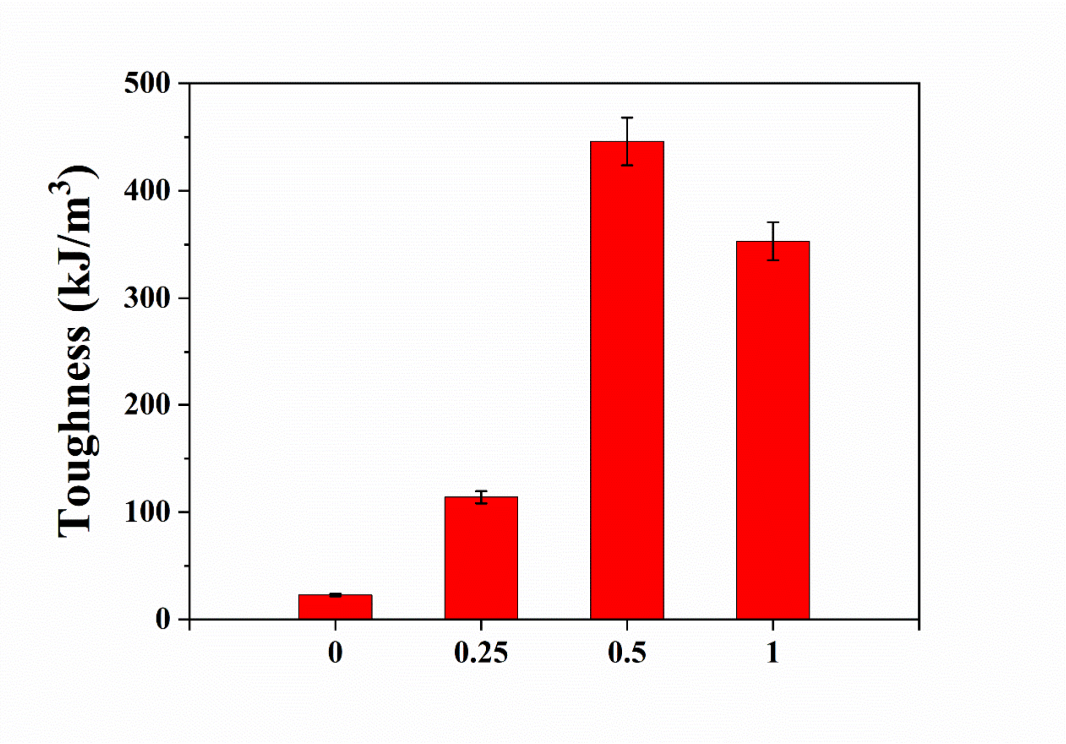

Figure S2. Toughness of P(AA-AMPS)-TA@BC-Ca^{2+} hydrogels with different concentrations of TA@BC (0/0.25/0.5/1) (the error bars show the error range of the experiment).

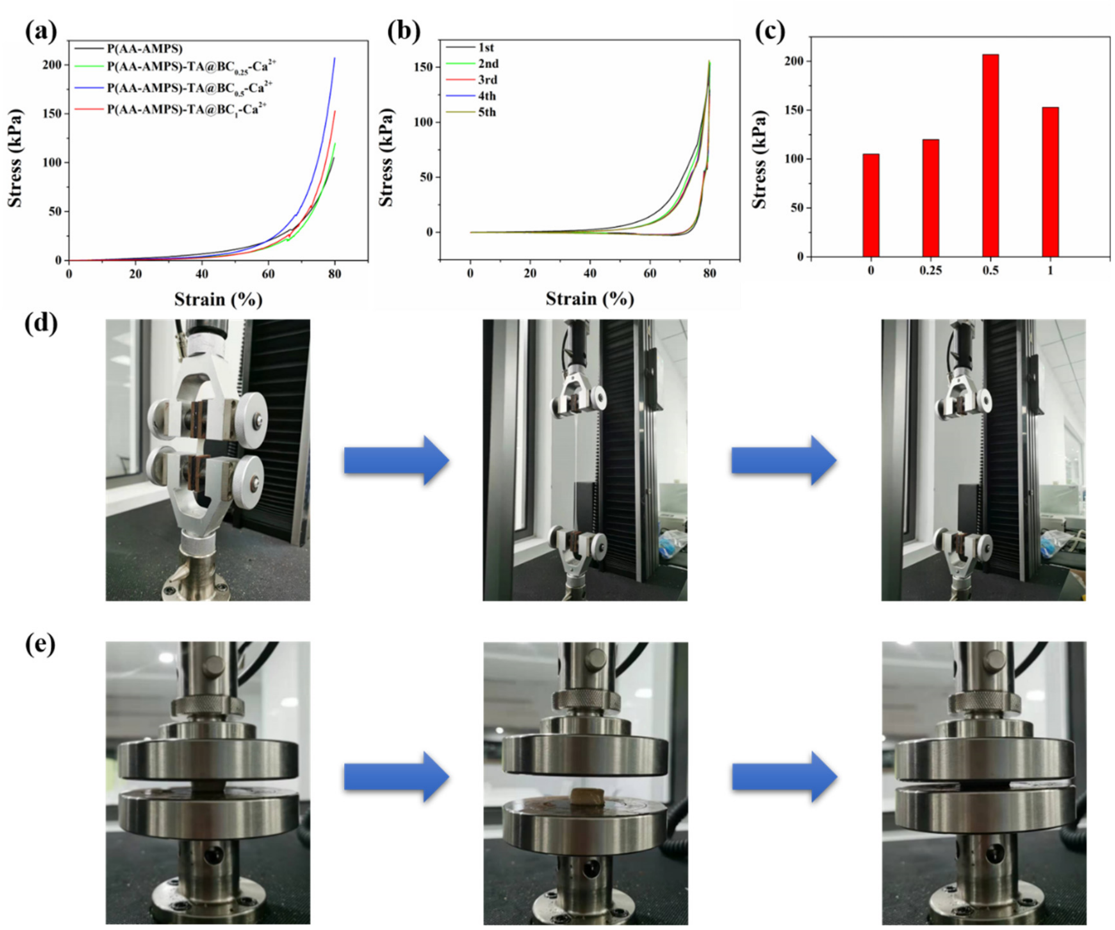

Figure S3. (**a**) Compressive stress-strain curves of P(AA-AMPS)-TA@BC-Ca^{2+} hydrogels with different concentrations of TA@BC; (**b**) Curves of compression-release cycles (80%, 5 cycles) of P(AA-AMPS)-TA@BC$_{0.5}$-Ca^{2+} hydrogel; (**c**) Compression stress-strain histogram of P(AA-AMPS), P(AA-AMPS)-TA@BC$_{0.5}$, P(AA-AMPS)-TA@BC$_{0.5}$-Ca^{2+} hydrogel; (**d**) Diagram of the stretching process; (e) Diagram of compression process.

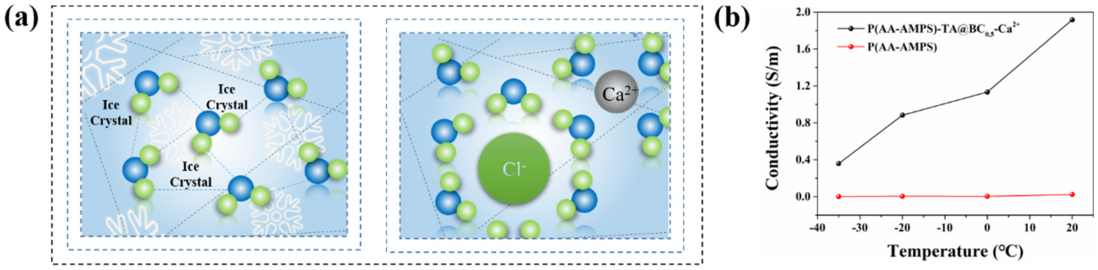

Figure S4. Mechanism of anti-freezing and moisturizing and electrical conductivity of hydrogel. (**a**) The interaction between Ca^{2+}/Cl^{-} ions and water molecules in $CaCl_2$ solution and the principle diagram of antifreeze and moisture retention (the unlabeled blue spheres represent oxygen atoms and the green spheres represent hydrogen atoms); (**b**) Electrical conductivity of P(AA-AMPS) and P(AA-AMPS)-TA@$BC_{0.5}$-Ca^{2+} hydrogels at different temperatures.

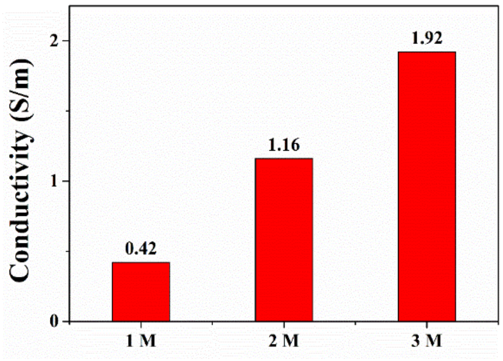

Figure S5. Hydrogel conductivity with diverse ion concentration

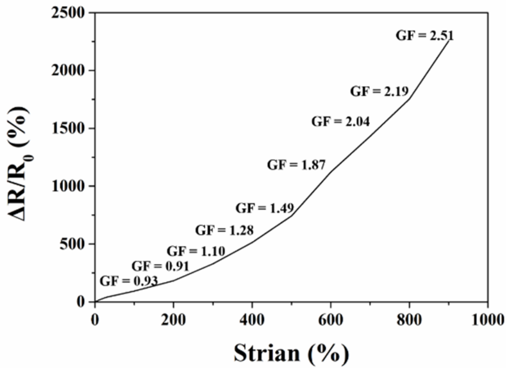

Figure S6. The RRC value and GF value of P(AA-AMPS)-TA@BC0.5-Ca^{2+} hydrogel in strain from 2.5% to 900%.

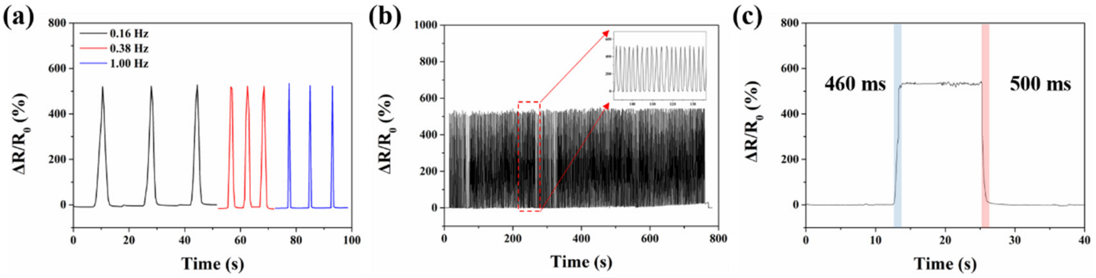

Figure S7. (**a**) Response frequency of P(AA-AMPS)-TA@BC0.5-Ca^{2+} sensor. (**b**) RRC of P(AA-AMPS)-TA@BC0.5-Ca^{2+} sensor elongated to a strain of 400% for 300 cycles under stretching mode. (**c**) Response and recovery time of P(AA-AMPS)-TA@BC0.5-Ca^{2+} strip under the 400% strain.
